# Deformable registration of chest CT images using a 3D convolutional neural network based on unsupervised learning

**DOI:** 10.1002/acm2.13392

**Published:** 2021-09-10

**Authors:** Yongnan Zheng, Shan Jiang, Zhiyong Yang

**Affiliations:** ^1^ School of Mechanical Engineering Tianjin University Tianjin China

**Keywords:** convolution neural network, data augmentation, deformable registration, unsupervised learning

## Abstract

**Purpose:**

The deformable registration of 3D chest computed tomography (CT) images is one of the most important tasks in the field of medical image registration. However, the nonlinear deformation and large‐scale displacement of lung tissues caused by respiratory motion cause great challenges in the deformable registration of 3D lung CT images.

**Materials and methods:**

We proposed an end‐to‐end fast registration method based on unsupervised learning, optimized the classic U‐Net, and added inception modules between skip connections. The inception module attempts to capture and merge information at different spatial scales to generate a high‐precision dense displacement vector field. To solve the problem of voxel folding in flexible registration, we put the Jacobian regularization term into the loss function to directly penalize the singularity of the displacement field during training to ensure a smooth displacement vector field. In the stage of data preprocessing, we segmented the lung fields to eliminate the interference of irrelevant information in the network during training. The existing publicly available datasets cannot implement model training. To alleviate the problem of overfitting caused by limited data resources being available, we proposed a data augmentation method based on the 3D‐TPS (3D thin plate spline) transform to expand the training data.

**Results:**

Compared with the experimental results obtained by using the VoxelMorph deep learning method and registration packages, such as ANTs and Elastix, we achieved a competitive target registration error of 2.09 mm, an optimal Dice score of 0.987, and almost no folding voxels. Additionally, the proposed method was much faster than the traditional methods.

**Conclusions:**

In this study, we have shown that the proposed method was efficient in 3D chest image registration. The promising results demonstrated that our method showed strong robustness in the deformable registration of 3D chest CT images.

## 1 INTRODUCTION

In deformable registration, a highly nonlinear, dense map is established between a pair of images. The deforming registration of 3D chest computed tomography (CT) images is critical to the application of radiotherapy, such as lung tumor motion tracking,^1^ brachytherapy,^2^ and dose planning.^3^ Although many researchers have worked on registration algorithms for chest CT images in recent decades,^4^ accurate and fast deformable registration of chest 3D CT images is still a challenging task.

3D chest CT images have been widely used in image‐guided radiation therapy^5^ to aid in treatment planning. The lung is a typical moving organ. In brachytherapy for lung cancer, doctors need to precisely pierce the puncture needle into the tumor and implant seeds. However, breathing and beating of the heart affect the expansion and contraction of the lungs, which will cause puncture errors. Therefore, it is essential to track the lung and estimate the precise dose needed before radiotherapy. In addition, because the brachytherapy plan for lung cancer is developed before surgery, introducing the precise preoperative plan into the surgery is an important measure for brachytherapy. However, due to the patient's breathing movement, some changes in the shape and position of the tumor during the preoperative and intraoperative period occurred. To calculate the effective dose distribution with lung movement, it is necessary to align the 3D chest CT images of different respiratory states to the 3D chest image of the reference state to track each voxel's dose. Deformable registration of 3D chest CT images is a feasible method for fast and accurate lung motion tracking.

Many existing state‐of‐the‐art methods for deformable registration use traditional algorithms, such as standard symmetric normalization (SyN)^6^ and diffeomorphic demons^7^ and free of deformations with b‐splines,^8^ to solve an optimization problem for each volume pair that aligns by using geometric methods. Generally, it is necessary to establish the displacement field via iterative optimization between images and tuning parameters precisely to solve this problem. These traditional algorithms are computationally expensive. Moreover, they learn nothing from registering each pair of images. Every time a new pair of images is registered, the traditional method reiterates and optimizes.

In recent years, researchers have tended to utilize learning‐based methods to improve the registration task. After training, the resulting network could register a pair of three‐dimensional medical images in seconds or several orders of magnitude faster than traditional methods. The accuracy of the resulting network registration of a pair of images can almost exceed that through the state‐of‐the‐art traditional methods. Image registration based on deep learning can be classified into two categories: supervised learning‐based methods and unsupervised learning‐based methods.

For supervised learning, several recent studies have obtained the dense ground‐truth displacement filled by geometry‐based methods^9^ or simulating deformation.^10^ However, the quality of the training data limits their performance. Hu et al.^11^ proposed using the segmented anatomical structure as the ground truth to train ConvNet. By using this method, ConvNet took fixed and moving image pairs as input and learned to align anatomical structures. Cao et al.^12^ also introduced the image similarity metric into ConvNet to help guide registration. Another supervised method that requires ground truth is to take the original image as a moving image, the original image warped by the simulated displacement field as a fixed image, and the simulated displacement field as the ground truth.^13^ Dubost et al.^14^ proposed automatically assessing the quality of registration to an atlas in clinical FLAIR MRI scans of the brain. The method applied a neural network‐based ventricle segmentation algorithm based on clinical FLAIR sequences to automatically assess the registration quality and validated the proposed quality assessment metric in a multiatlas registration framework. Although supervised learning has great potential in the field of image registration, it is cumbersome to acquire ground truth via traditional registration tools.

Compared to supervised learning, the registration method based on supervised learning must provide ground truth corresponding to the training samples when training the network. These annotations must be manually marked by professional radiologists, and this process is very time‐consuming and laborious. Registration based on unsupervised learning does not require additional manually generated or labeled ground truth (GT), which reduces the complexity of the registration process. For unsupervised learning approaches, such as recently published VoxelMorph,^15^ DLIR,^16^ and FAIM^17^ methods, performance was comparable to those of traditional methods. The registration process of these unsupervised learning methods is roughly the same. A pair of image pairs was fed into the ConvNet, and the similarity measured between images was considered to be part of the loss function. At the same time, gradient back propagation could be operated by differentiable warping. Balakrishnan et al.^18^ proposed an end‐to‐end registration method of 3D medical images with UNet as the core based on unsupervised learning. They defined registration as a parametric function and optimized its parameters, given a set of images from a collection of interest. Based on their previous work,^15^ they added auxiliary segmentation of each anatomical structure in the training data to guide the training. They also proposed a probabilistic generation model and derived an inference algorithm based on unsupervised learning, which could not only provide diffeomorphic guarantees and uncertainty estimates but also ensure that a pair of images can be accurately registered.^19^ Zhang et al.^20^ presented an encoder‐decoder network for the evaluation of the stationary velocity field to perform diffeomorphic registration, which improved the invertibility of the deformation field. Xu et al.^21^ achieved better performance by predicting a dense displacement field using a cascade registration subnetwork. The methods mentioned above have achieved good results on brain and liver medical images. DLIR^16^ used the unsupervised learning‐based method but could only estimate a sparse displacement field interpolated by a third‐order b‐spline kernel.

In this study, we proposed a ConvNet method based on unsupervised learning for deformable registration of 3D chest CT images, which could directly predict the 3D dense displacement field. This architecture captured the feature maps of multiscale UNet.^22^ We also learned from GoogLeNet^23^ of the multidimensional convolution kernel and used different sizes of convolution kernels to improve the feature perception ability of the network. The limitation of less training data was solved by artificially generated 3D chest CT images. To suppress irreversible deformation, the negative Jacobian determinant of the displacement field was used as the penalty loss. Experiments show few folding voxels in the resulting warped image. When ConvNet was trained, it could register a pair of unseen 3D chest CT images in one shot.

## 2 MATERIALS AND METHODS

We proposed a ConvNet method to estimate an optimal parameterized mapping function ϕ directly for the image pair (e.g., a moving image $I_{m}$ and a fixed image $I_{f}$). The mapping function ϕ is a dense nonlinear correspondence of all voxels between the moving image $I_{m}$ and fixed image $I_{f}$. The warped image $I_{m}\circ \phi$ from a moving image $I_{m}$ can be aligned to a fixed image $I_{f}$. In our method, the global mapping function ϕ(x)=x+s(x) is formed by an identity transform and dense displacement field s. When ConvNet is trained, we can obtain the displacement field s from an unseen image pair.

**FIGURE 1:**
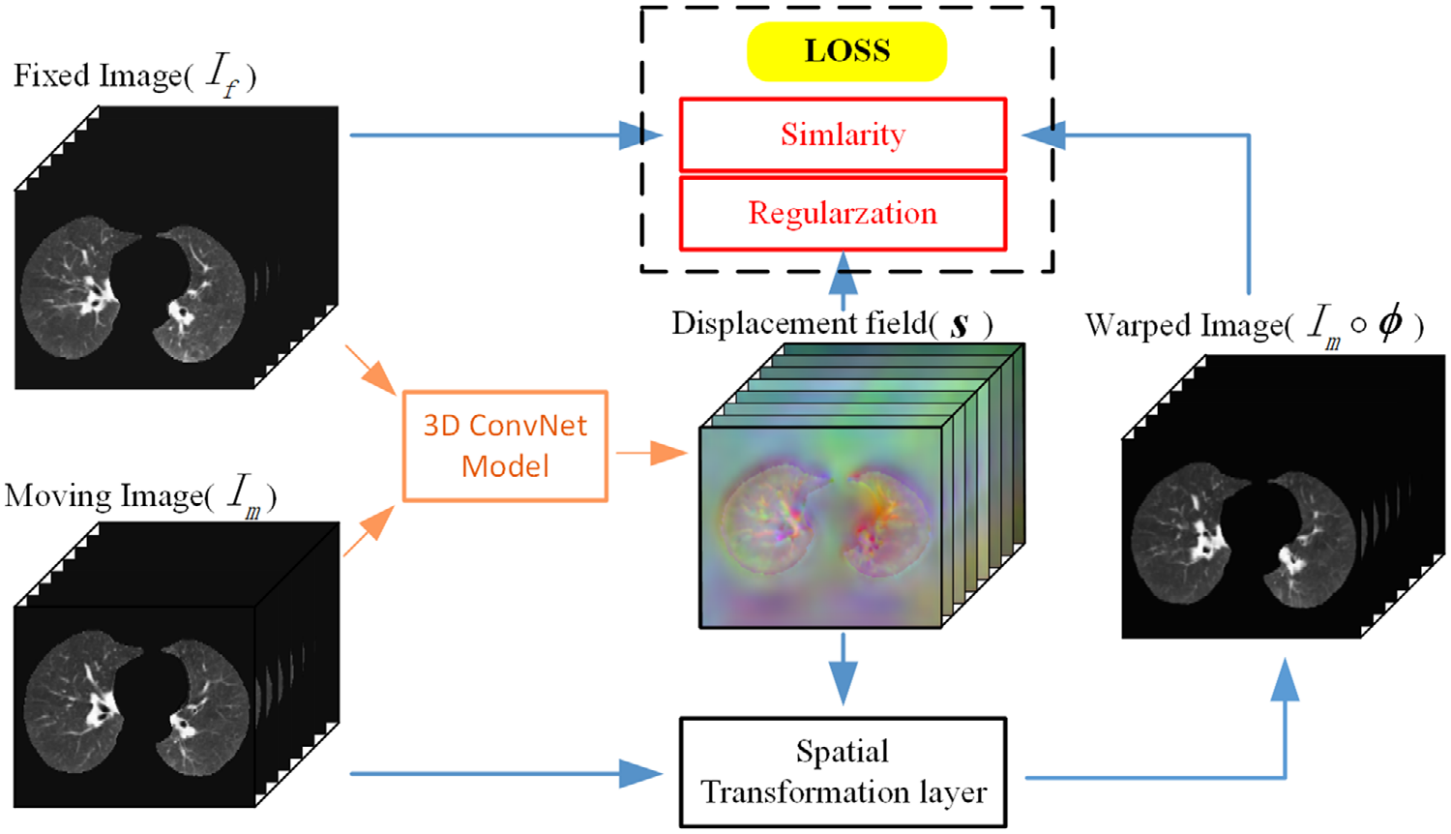
An overall diagram of our method

As shown in Figure 1, ConvNet is designed in the end‐to‐end form. During training, a pair of 3D chest CT images ($I_{m}$ and $I_{f}$) are concatenated into a two‐channel 3D image and fed into ConvNet. The convolutional layers of ConvNet calculate s. We use a spatial transformation layer to warp $I_{m}$ into $I_{m}\circ \phi$. By taking the similarity of $I_{f}$ and $I_{m}\circ \phi$ as a part of the loss function, the network parameters are continuously optimized during training. The displacement field is punished by regularization terms to encourage smoothness.

### 2.1 Preprocessing

We focused on the registration performance in lung fields. The lung fields were directly extracted from the EMPIRE10,^24^ DIR‐Lab 4DCT,^25^ and POPI^26^ datasets. We cropped the entire lung field and resampled all images to a size of 224×144×192. The image voxel values were normalized to [0, 1]. Finally, affine registration was performed on intrapatient images (CT images of the same patient in different periods).

### 2.2 Data augmentation

To overcome the problem of overfitting caused by having only small amounts of training data, training data were augmented by the thin plate spline (TPS) transformation. The original STN experimented on three specific transformation forms: affine transformation, projective transformation, and TPS. Compared with the affine transformation, projective transformation, and TPS, TPS could perform more abundant deformation on images. Therefore, 3D TPS was used to augment our 3D chest CT images.

We denoted a 3D grid in the space of the simulated image and real image. The size of the 3D grid was the same as the 3D chest CT image with N grid points. We also denoted N pairs of the corresponding points $m_{i}=\left(x_{i},y_{i},z_{i}\right)$ and points $n_{i}=\left(x_{i}^{\prime },y_{i}^{\prime },z_{i}^{\prime }\right)$. Here, $m_{i}$ was a control point on the grid points of the simulated image, and $n_{i}=m_{i}+u\left(-h,h\right)$ was a control point on the real image. Additionally, $u\left(-h,h\right)\in R^{1\times 3}$ obeys a uniform probability distribution between (−h,h). The 3D TPS interpolation function is as follows:

(1) $$\begin{array}{ccc} & & \left[f\left(x,y,z\right)=\left(\begin{array}{c} a_{1x}\\ a_{1y}\\ a_{1z} \end{array}\right)+\left(\begin{array}{c} a_{2x}\\ a_{2y}\\ a_{2z} \end{array}\right)x+\left(\begin{array}{c} a_{3x}\\ a_{3y}\\ a_{3z} \end{array}\right)y+\left(\begin{array}{c} a_{4x}\\ a_{4y}\\ a_{4z} \end{array}\right)z\right.\\ & & \;\;\left.+\sum_{i=1}^{n}\left(\begin{array}{c} w_{\mathrm{ix}}\\ w_{\mathrm{iy}}\\ w_{\mathrm{iz}} \end{array}\right)U\left(\left|m_{i}-\left(x,y,z\right)\right|\right)\right], \end{array}$$

where U(r) is a radial basis function. We use $U\left(r\right)=r^{2}\log\left(r\right)$. We defined $r_{i,j}=\left|m_{i}-n_{j}\right|$, which represented the Euclidean distance between $m_{i}$ and $n_{j}$. The matrix and vectors can be defined as follows:

(2) $$K={\left[\begin{array}{c} U\left(r_{1,1}\right)\cdots U\left(r_{1,N}\right)\\ \;\;\;\;\;\vdots \ddots \;\;\;\;\;\vdots \\ U\left(r_{N,1}\right)\cdots U\left(r_{N,N}\right) \end{array}\right]}_{N\times N},$$

The parameters of the function f(x,y,z) can be obtained by the following linear equation:

(3) $$\left\{\begin{array}{c} \mathrm{Kw}+\mathrm{Pa}=V\\ P^{T}w=0 \end{array}\right.,$$

where P and V are the matrices of *N* points in the real and simulated images, respectively.

First, the coordinate mapping between N pairs of corresponding points in simulated images and real images can be obtained by using a 3D TPS interpolation function f(x,y,z). Then, the gray value of each point of the real images is interpolated into the simulated images.

**FIGURE 2:**
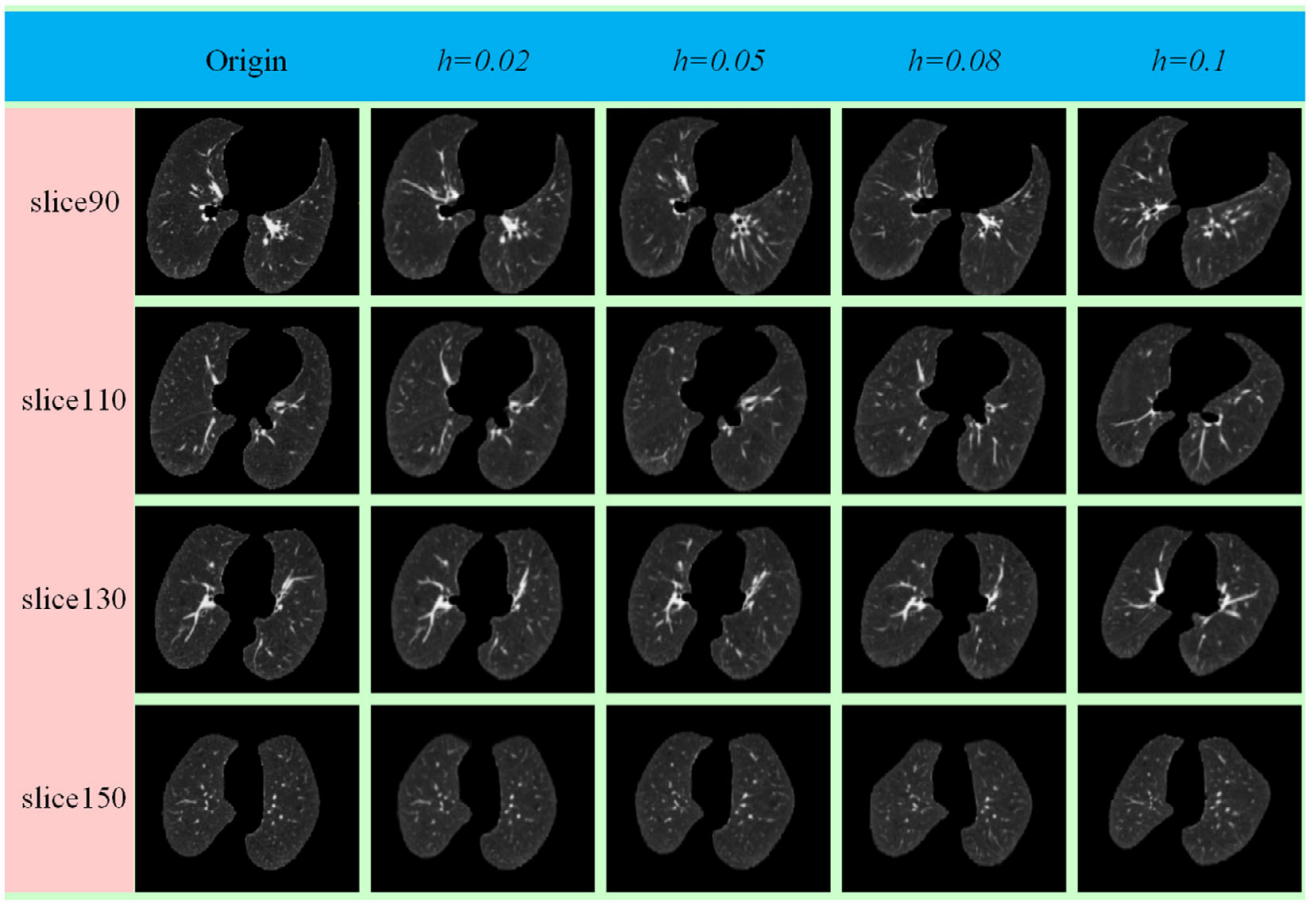
Examples of augmented data of 3D chest CT images from the EMPIRE10 dataset. From top to bottom, the different slices of the axial plane are shown. From left to right, the original images and the transformed images with different scales are shown

In our data augmentation experiment, simulated images were generated by setting *N *= 5^3^, *h *= 0.02; *N *= 5^3^, *h *= 0.0^5^; *N *= 5^3^, *h *= 0.08; and *N *= 5^3^, *h *= 0.1. We set *h* between 0.02 and 0.1 to avoid overstretching the images. Examples are available in Figure 2. It is possible to augment the training data using a 3D TPS transformation method.

### 2.3 ConvNet architecture

**FIGURE 3:**
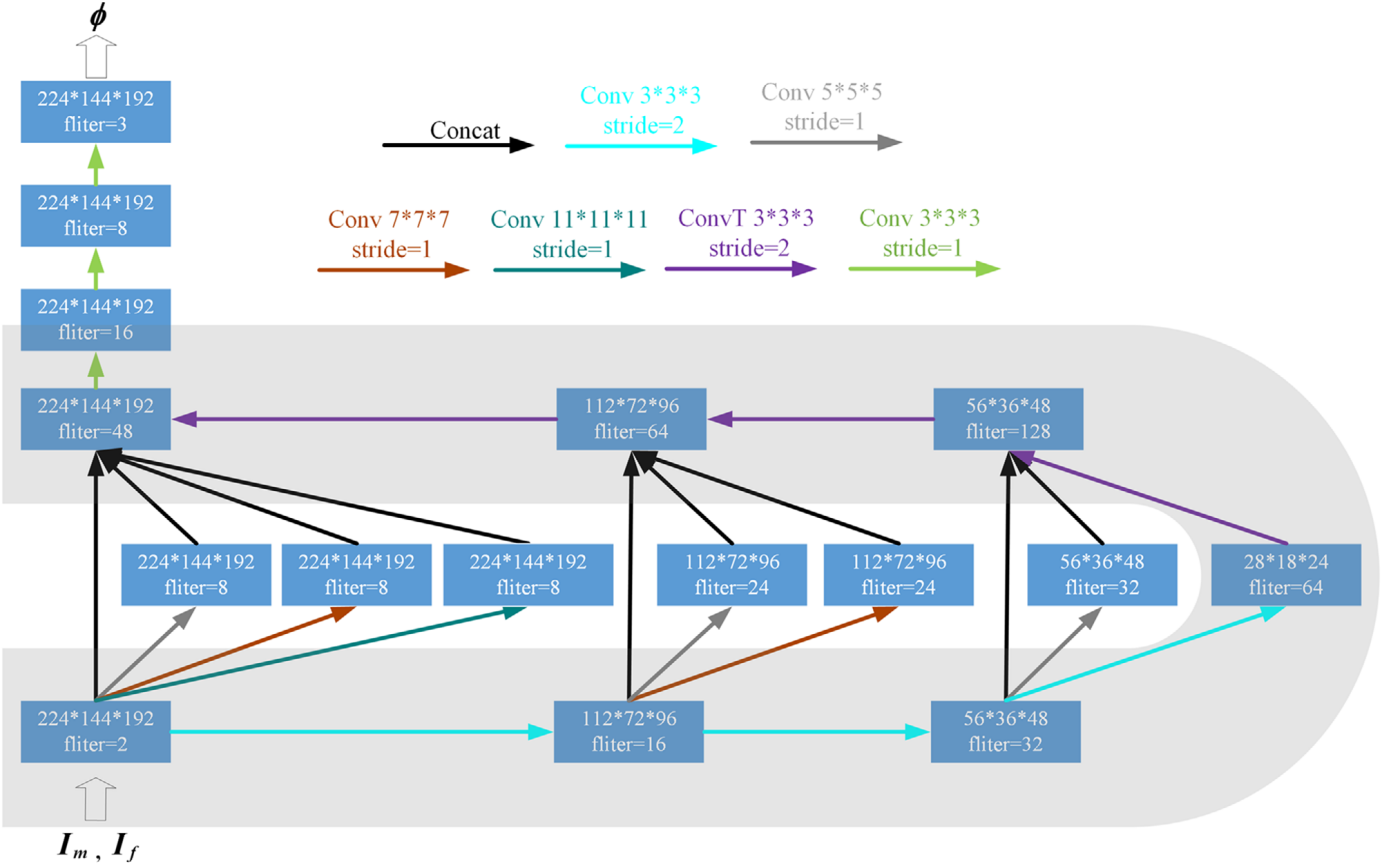
ConvNet architecture. Each rectangle represents a 3D volume, and the number of filters (channels) is marked on the rectangle

As shown in Figure 3, our ConvNet architecture is similar to UNet. The main architecture of the network is composed of an encoder‐decoder with skip connections. The inception modules are inserted in the middle of the “U” structure. The inception modules are composed of convolutional layers with a convolutional kernel of different sizes.

ConvNet takes a two‐channel 3D chest CT image that is formed by concatenating $I_{f}$ and $I_{m}$ as input. Note that both $I_{f}$ and $I_{m}$ are from intrapatient. The input size of the proposed ConvNet is 224×144×192×2. LeakyReLU activations are applied to the end of each 3D convolutional block except for the last layer. The last layer of our ConvNet uses linear activation, which ensures that any value (positive and negative) in the displacement field is possible. In the encoder, convolutional kernel stride = 2 is used three times to reduce the spatial dimensions of the volume to (1/2)^3^. The operation of continuous downsampling convolution is similar to the operation of traditional image pyramid extraction. Each volume to downsample goes through the inception module. The inception module attempts to capture and merge information at different spatial scales to generate the displacement field. In the decoder, transposed convolutional layers are used three times for upsampling. There are three concatenating skip connections between the encoder and the decoder. In both the encoder and the decoder, the convolution kernel size is 3×3×3. The displacement field of size 224×144×192×3 is output at the end of the ConvNet.

### 2.4 Spatial transformation layer

The spatial transformation layer is inspired by spatial transformer networks (STN).^27^ The purpose of the spatial transformation layer is to compute $I_{m}\circ \phi$. The position of each voxel in $I_{m}$ is calculated in the space of $I_{f}$. This operation means that we use ϕ to warp $I_{m}$ and then obtain $I_{m}\circ \phi$. Since the position of the voxel is indexed as an integer, we linearly interpolate the voxel value of $I_{m}$ at eight neighborhood voxels in the X, Y, and Z directions. Because the operations of linear interpolation ensure that the spatial transformation layer is differentiable, the errors could be back‐propagated during optimization.

### 2.5 Loss function

The loss function is composed of three components: $L_{\mathrm{sim}}$, $R_{\mathrm{jac}}$, and $R_{\mathrm{der}}$. $L_{\mathrm{sim}}$ penalizes the difference in appearance. Both $R_{\mathrm{jac}}$ and $R_{\mathrm{der}}$ are regularization terms that can help predict smooth registration fields. $R_{\mathrm{jac}}$ penalizes transformations that have negative Jacobian determinants. $R_{\mathrm{der}}$ penalizes the first derivative of s to predict a smooth registration field.

We set $L_{\mathrm{sim}}$ to the negative local normalized cross‐correlation coefficient of $I_{f}$ and $I_{m}\circ \phi$. $\tilde{I_{f}}\left(p\right)$ and $\left[\tilde{I_{m}}\circ \phi \;\right]\left(p\right)$ are defined as the image intensities with local mean intensities subtracted out. We calculate the local image mean intensities and normalized cross‐correlation coefficient over a volume of 11×11×11. The local normalized cross‐correlation coefficient is defined as follows:

(4) $$\begin{array}{ccc} & & \left[\mathrm{NCC}\left(I_{f}\;,I_{m}\circ \;\phi \right)=\sum_{p\in \Omega }\frac{{\left(\sum_{p_{i}}\left(I_{f}{\left(p\right)}_{i}-\tilde{I_{f}}\left(p\right)\right)\left(\left[I_{m}\circ \;\phi \;\right]\left(p_{i}\right)-\left[\tilde{I_{m}}\circ \phi \;\right]\left(p\right)\right)\right)}^{2}}{\left(\sum_{p_{i}}{\left(I_{f}\left(p_{i}\right)-\tilde{I_{f}}\left(p\right)\right)}^{2}\right)\left(\sum_{p_{i}}{\left(\left[I_{m}\circ \;\phi \;\right]\left(p_{i}\right)-\left[\tilde{I_{m}}\;\circ \phi \;\right]\left(p\right)\right)}^{2}\right)}\right] \end{array}$$

Here, $p_{i}$ iterates over an 11×11×11 volume. Since higher normalized cross correlation (NCC) values represent better alignment, we define $L_{\mathrm{sim}}$ to be:

(5) $$L_{\mathrm{sim}}\left(I_{f},I_{m}\circ \;\phi \right)=1-\mathrm{NCC}\left(I_{f},I_{m}\circ \phi \right)$$

A discontinuous s will lead to the existence of a folding voxel in $I_{m}\circ \phi$. The Jacobian determinant of the displacement field is negative, where the warped image is folding. We set the Jacobian determinant for each point p(i,j,k) in s as:

(6) $$\det\left(\nabla s(p)\right)=\left|\begin{array}{c} \begin{array}{ccc} \frac{\partial i}{\partial x} & \frac{\partial j}{\partial x} & \frac{\partial k}{\partial x} \end{array}\\ \begin{array}{ccc} \frac{\partial i}{\partial y} & \frac{\partial j}{\partial y} & \frac{\partial k}{\partial y} \end{array}\\ \begin{array}{ccc} \frac{\partial i}{\partial z} & \frac{\partial j}{\partial z} & \frac{\partial k}{\partial z} \end{array} \end{array}\right|,$$

where the Jacobian determinant is ≤ 0, which means that the warped image folding occurred. To avoid folding, the regularization term is used to constrain the deformation, which is defined as:

(7) $$\left[R_{\mathrm{jac}}\left(s\right)=\sum_{p\in \Omega }\left(\left|\det\left(\nabla s\left(p\right)\right)\right|-\det\left(\nabla s\left(p\right)\right)\right)\right]$$

When the Jacobian determinants of the displacement field are all positive, $R_{\mathrm{jac}}$ is equal to 0 and will not contribute to the total loss. At the same time, $R_{\mathrm{jac}}$ will be activated by any transformations that have a negative determinant. We also use a regularization term $R_{\mathrm{der}}$ related to the first derivative of the displacement field. By punishing the first derivative of the displacement field, the overall smoothness of the predicted displacement field is constrained. Balakrishnan et al.^18^ discussed the effect of the regularization term. The expression of $R_{\mathrm{der}}$ can be described as:

(8) $$R_{\mathrm{der}}\left(s\right)=\sum_{p\in \Omega }{\left\|\nabla s(p)\right\|}^{2}$$

The total training loss is defined as:

(9) $$L_{\mathrm{total}}\left(I_{f},I_{m}\circ \phi \right)=L_{\mathrm{NCC}}\left(I_{f},I_{m}\circ \;\phi \right)+\beta R_{\mathrm{jac}}\left(s\right)+\alpha R_{\mathrm{der}}\left(s\right),$$

where α and β are hyperparameters that control the degree of regularization.

### 2.6 Implementation details

In the training stage, the size of the 3D chest CT images was 224×144×192 because of GPU memory limitations. To alleviate the overfitting problem, data augmentation of training data was conducted out using a 3D TPS‐based method. The Adam optimizer was used to optimize the learning model. After many experiments, we found that the model with a learning rate of 10^–4^ had the best effect. In this work, the learning model was trained for 20 epochs. Considering the GPU memory limitation, the batch size was set to 1. The regularization ability of the learning model was improved by using batch normalization. The other hyperparameters α were set to 1, and β was set to $10^{-5}--10^{-2}$.

During training, moving images were resampled to warped images by using linear resampling. When evaluating the Dice score, lung masks were resampled by using nearest neighbor resampling. The proposed method was implemented by Keras with a Tensorflow backend by using an NVIDIA TITAN Xp 12 GB GPU.

### 2.7 Datasets

The proposed method used three chest CT image datasets: EMPIRE10, DIR‐Lab 4DCT, and POPI. All patients underwent contained chest 3D CT of the intrapatient region at different times.

The DIR‐Lab 4DCT dataset consists of 10 4D chest CT scans, which are 10‐time points in a full breathing cycle, for a total of 100 scans. We used the data of the two phases with the largest relative deformation: maximum exhalation and maximum inhalation. For each 4D scan, 300 manually identified anatomical landmarks were annotated in each maximum exhalation and inhalation scan. Binary lung masks were also provided for each scan.

The EMPIRE10 dataset consists of 30 pairs of 3D chest CT scans, and each pair of scans is taken as intrapatient. Binary lung masks are also provided from each scan.

The POPI dataset consists of six 4D chest CT scans, and each 4D chest CT image consists of 10 3D chest CT scans that represent 10 different phases of a full breathing cycle. We used the data of the two phases with the largest relative deformation: maximum exhalation and maximum inhalation. For each 4D scan, 100 manually identified anatomical landmarks were annotated in each maximum exhalation and inhalation scan.

### 2.8 Experimental setup

We used 30, 6, and 10 pairs of 3D CT scans from EMPIRE10, POPI, and DIR‐Lab 4DCT as training, validation, and test sets, respectively. The data augmentation method based on 3D TPS transformation was used to increase the diversity of the data. For each image pair in the training and validation sets, we created 100 pairs of different simulated scans (25 for *h *= 0.02, 25 for *h *= 0.05, 25 for *h *= 0.08, and 25 for *h *= 0.1; all of them *N* = 5^3^). In total, we had 6060, 1212, and 20 chest CT scans for the training, validation, and test sets, respectively.

### 2.9 Evaluation metrics

Since lungs tend to occupy many voxels, the alignment of the lung surfaces is evaluated using the target registration error (TRE) of distinct landmarks:

(10) $$\mathrm{TRE}=\frac{1}{n}\sum_{i=1}^{n}{\left\|x_{f}\circ \phi -x_{m}\right\|}_{2},$$

where $x_{m}$ and $x_{f}$ are the set of corresponding landmarks in the moving image and fixed image, respectively.

Dice is not a great measure for how well the surfaces of the lungs align, but it can be used to evaluate the volume overlap of the lung fields. The mask of the moving image is warped by the predicted registration field. Then, the Dice score of the warped mask with the ground‐truth mask of the fixed image is computed. The Dice score can be expressed as:

(11) $$\mathrm{Dice}\left(A_{M\circ \phi },A_{F}\right)=\frac{2\left|A_{M\circ \phi }\cap A_{F}\right|}{\left|A_{M\circ \phi }\right|+\left|A_{F}\right|},$$

where $A_{M\circ \phi }$ and $A_{F}$ are the voxel sets in the warped mask and the mask of the fixed image, respectively.

Additionally, image folding is anatomically implausible. The number of folding voxels could be counted by the Jacobian determinant of the displacement field. Our algorithm was also evaluated by using the Jacobian determinant of the displacement field (described in Section 2.5).

### 2.10 Baseline methods

The proposed method was compared with the publicly available ANTs^28^ toolkit using SyN. A cross‐correlation similarity measure was used to guide the registration. We used the ANT smoothness parameters, including a SyN step size of 0.25 and Gaussian parameters (3, 0), at four scales with at most 219 iterations each.

Our method was also compared with the B‐spline registration method based on the Elastix^29^ toolkit. We used normalized cross‐correlation as the similarity measure, using five resolutions of 1000 iterations.

We also compared our approach with VoxelMorph, a fast learning‐based algorithm. VoxelMorph proposed two network structures, Vm1 and Vm2, both of which were based on normal U‐Net networks. We experimented on both Vm1 and Vm2. The original VoxelMorph was trained on brain registration. We retrained VoxelMorph on the EMPIRE10 dataset to make the comparison fair.

## 3 RESULTS

Ten pairs of scans from DIR‐Lab 4DCT were used as a test set to evaluate the method. In the experimental results diagram, we used the weights of different Jacobian regularization terms to conduct the experiments, which were represented by “*β *=$\;10^{-2},$” “*β *=$\;10^{-3},$” “*β *=$\;10^{-4},$” and “*β *=$\;10^{-5}\;.$” The validation set was used to estimate the generalization error in the training and to update the hyperparameters. The results were reported on the test dataset.

**FIGURE 4:**
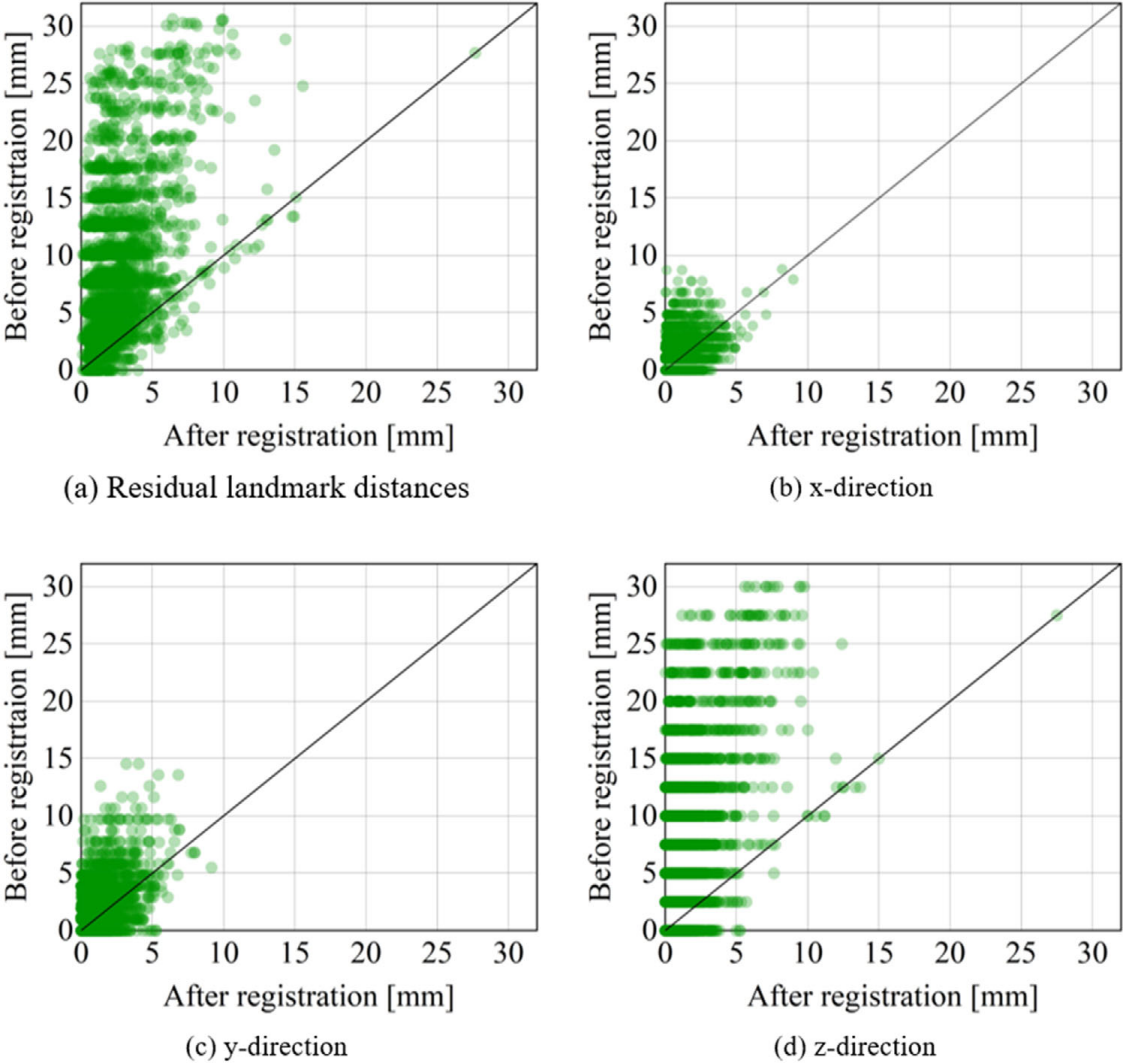
Residual distance errors. (a) Shows scatterplots of the global residual distance. (b)–(d) show the residual distance along the x‐, y‐, and z‐axes, respectively. Darker colors express a higher density of points

The test dataset contained 3000 corresponding landmarks. All of the available corresponding landmarks were used to calculate TRE. The results of the TRE are shown in Table 1 and Figure 4. Our method achieved a promising minimum mean registration error of 2.09 mm with a standard deviation of 1.55. ANTs (SyN) overall gave the best performance, but our method was better than the others.

**TABLE 1:** List for TRE of the intrapatient of 10 3D chest CT images from the DIR‐Lab 4DCT

Scan	Initial	Elastix (BSpline)	ANTs (SyN)	Vm1	Vm2	*β *= 10^‐3^	*β *= 10^‐4^	*β *= 10^‐5^
Case 1	3.89 (2.78)	1.08 (0.70)	1.09 (0.75)	1.92 (1.35)	1.69 (1.13)	1.48 (0.96)	1.28 (0.86)	1.12 (0.83)
Case 2	4.33 (3.90)	1.14 (0.67)	1.04 (0.67)	2.10 (1.42)	1.36 (1.67)	1.95 (1.23)	1.22 (0.79)	1.21 (0.77)
Case 3	6.94 (4.05)	1.21 (0.81)	1.18 (0.78)	2.54 (2.01)	1.23 (1.02)	2.70 (1.69)	1.61 (1.05)	1.49 (0.98)
Case 4	9.83 (4.85)	1.69 (1.15)	1.50 (1.04)	3.75 (2.62)	1.68 (1.31)	3.18 (2.03)	2.17 (1.54)	1.83 (1.23)
Case 5	7.47 (5.50)	1.75 (1.30)	1.43 (1.19)	3.01 (2.84)	1.77 (2.42)	3.16 (2.11)	1.98 (1.49)	1.75 (1.26)
Case 6	10.89 (6.96)	2.06 (2.12)	1.50 (1.06)	4.00 (3.11)	2.88 (2.16)	3.13 (2.06)	2.61 (1.71)	2.50 (1.65)
Case 7	11.02 (7.42)	1.96 (1.52)	2.35 (1.93)	5.58 (3.97)	3.54 (3.65)	5.39 (3.41)	4.38 (2.88)	3.30 (2.34)
Case 8	14.99 (9.00)	1.87 (1.64)	1.51 (1.37)	5.60 (3.81)	4.45 (3.64)	6.26 (3.58)	3.25 (2.36)	2.86 (2.41)
Case 9	7.91 (3.97)	1.46 (1.31)	1.83 (1.27)	3.58 (2.62)	2.56 (2.23)	3.31 (2.00)	3.25 (2.05)	2.76 (2.05)
Case 10	7.30 (6.34)	1.75 (1.62)	1.42 (1.00)	4.44 (3.10)	2.03 (1.76)	3.56 (2.59)	2.41 (1.71)	2.04 (1.22)
Total	8.46 (6.58)	1.60 (1.28)	1.49 (1.16)	3.65 (2.53)	2.35 (1.81)	3.41 (2.37)	2.42 (1.78)	2.09 (1.55)

Note: The mean (standard deviation) of TRE (mm) is in millimeters. Due to space constraints, β = 10–^2^ is omitted.

**FIGURE 5:**
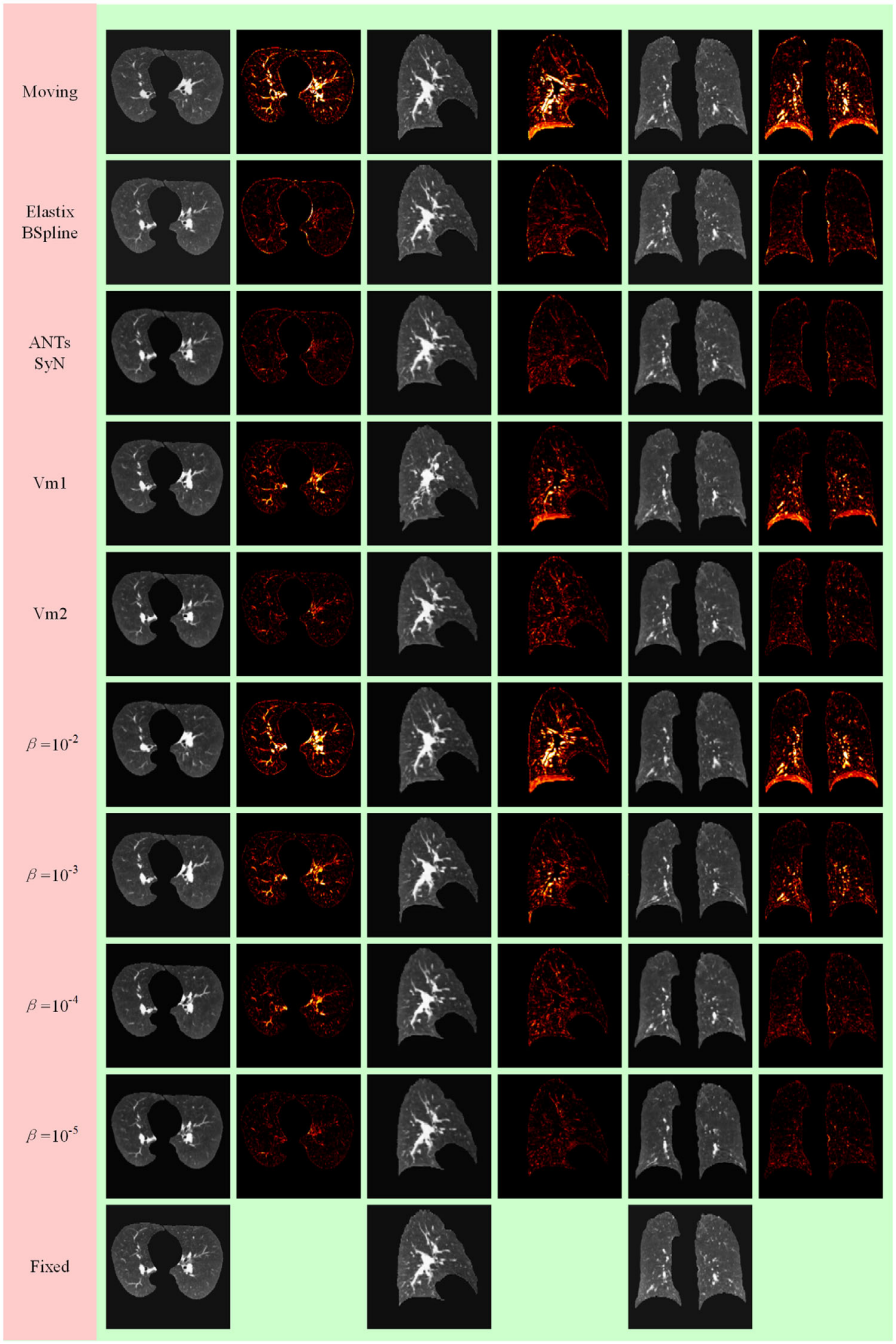
Example results of the intrapatient 3D chest CT from the DIR‐Lab 4DCT. The columns show axial, sagittal, and coronal slices within between corresponding heatmaps of the absolute difference with respect to the fixed image

Figure 5 shows the registration results of aligning two images from the test dataset. The absolute difference was calculated by the warped image and fixed image, which could qualitatively evaluate the quality of the deformation inside the lungs. In this work, different alignment accuracies could be obtained by adjusting *β* in the loss function. Visually, our method achieved promising results.

**FIGURE 6:**
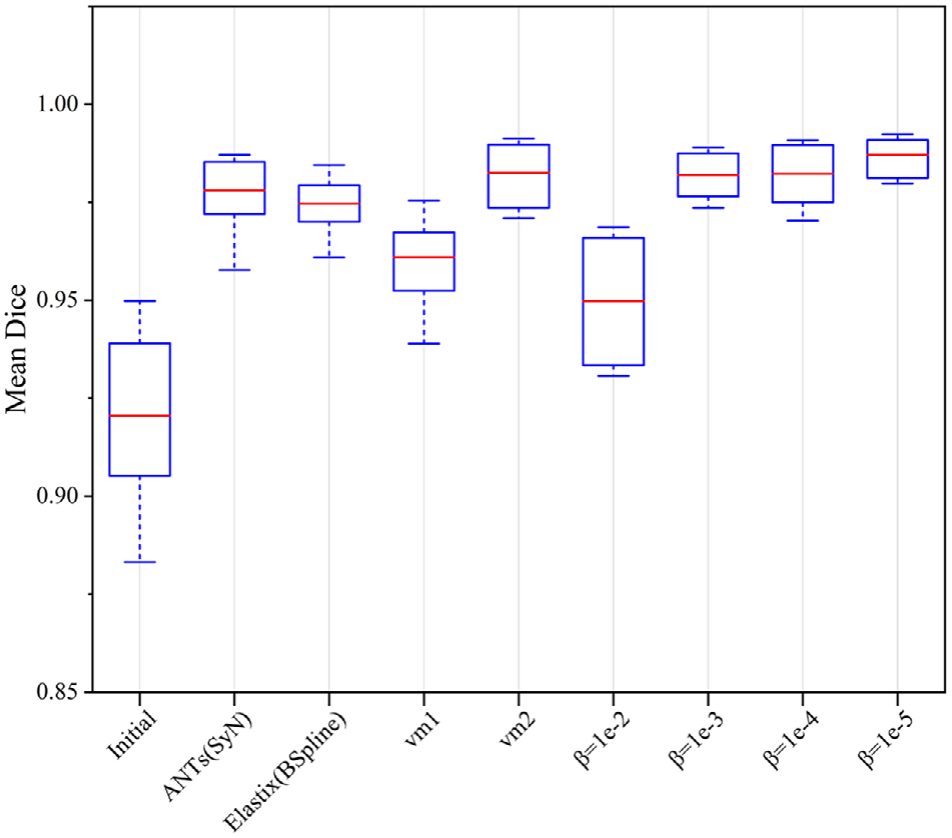
Mean Dice scores on 10 intrapatient chest CT images from DIR‐Lab 4DCT for our methods, VoxelMorph, and traditional methods

Figure 6 shows the mean Dice scores of all methods on the test dataset. We calculated Dice scores for lung fields of a 3D chest CT image and computed the mean across the test dataset. The experimental results showed that the registration performance of our method is better than that of VoxelMorph and SyN.

**FIGURE 7:**
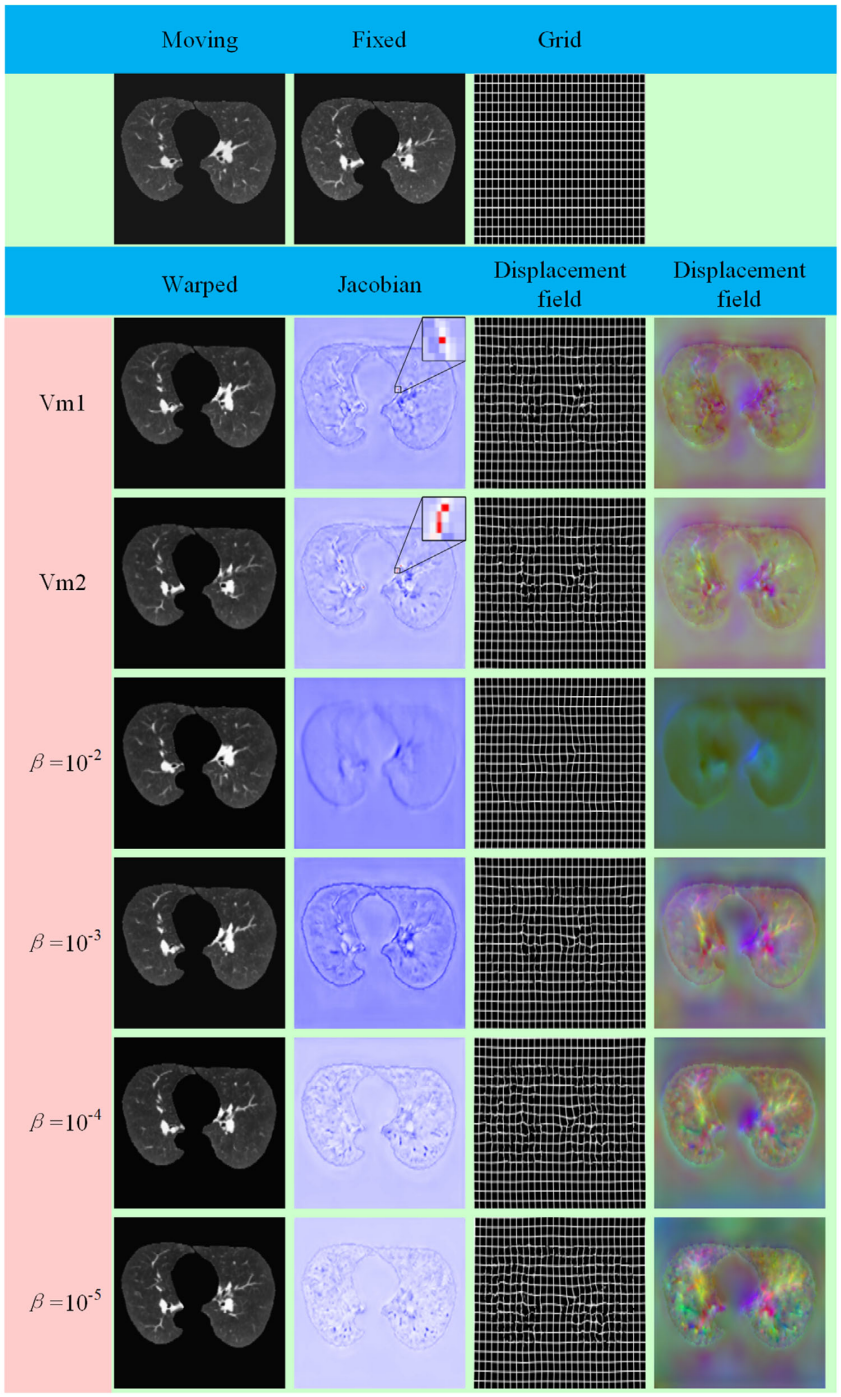
Example 3D chest CT image slice of resulting images for our methods and VoxelMorph. In the Jacobian determinant images of the displacement field, locations that represent singularity are marked in red. A brighter color indicates a larger warping in an RGB image

The representative results are shown in Figure 7. The displacement fields were presented as RGB images. The three channels of the RGB image correspond to the three dimensions of the displacement field. Grid images warped by the displacement field are also given in Figure 7. It is worthwhile to note that the proposed method and VoxelMorph used the same transform standard, but they were different from those of ANTs and Elastix. The resulting folding of all of the methods was also displayed in Jacobian images.

Table 2 gives a summary of all registration results. The execution time required for all methods on the GPU and CPU was also provided. The execution time of our method was much faster than that of traditional methods. To date, there have been no GPU implementations for ANTs and Elastix. The parameters of our ConvNet were slightly less than vm2. Our method gained almost no folding while achieving high registration accuracy.

**TABLE 2:** Results of the 10 intrapatient 3D chest CT images from the DIR‐Lab 4DCT

	Mean Dice	Folding	TRE	CPU sec	GPU sec	Parameters
Initial	0.921 (0.201)	–	8.46 (6.58)	–	–	–
Elastix (BSpline)	0.975 (0.008)	69 979 (81 495)	1.60 (1.28)	238.6 (4.8)	–	–
ANTs (SyN)	0.978 (0.008)	50 (137)	1.49 (1.16)	12 582 (1321)	–	–
Vm1	0.961(0.011)	537 (431)	3.65 (2.53)	7.2 (0.3)	2.8 (0.6)	116 971
Vm2	0.980 (0.007)	3522 (2978)	2.35 (1.81)	21.5 (1.2)	3.7 (0.6)	277 875
*β *= 10^‐2^	0.950 (0.015)	0	5.62 (4.87)	23.6 (0.4)	3.5 (0.7)	250 861
*β *= 10^‐3^	0.982 (0.006)	0	3.41 (2.37)	23.6 (0.4)	3.5 (0.7)	250 861
*β *= 10^‐4^	0.982 (0.008)	13 (3)	2.42 (1.78)	23.7 (0.5)	3.4 (0.7)	250 861
*β *= 10^‐5^	0.987 (0.005)	59 (5)	2.09 (1.55)	23.6 (0.9)	3.5 (0.7)	250 861

Note: All of the experimental results are given as the mean (standard deviation).

## 4 DISCUSSION

In this study, we developed a new unsupervised learning method for 3D chest CT image registration. We exploited NCC between moving and fixed image pairs to train a ConvNet. The ground truth of the image registration is not required. Large‐scale training data are generated by a 3D TPS‐based data augmentation method. The Jacobian regularization term is added to the loss function and effectively reduces the folding of the warped image. Experimental results confirmed that the proposed method achieved promising results with a TRE of 2.09 mm and a Dice score of 0.987, with very little folding. The proposed methodology has been proven to be robust in the 3D chest CT lung image registration task.

It is well known that deep learning methods are driven by a large amount of training data. A few 3D chest CT images of intrapatient patients were selected from the EMPIRE10 dataset as the origin materials of the training set. The original dataset is very limited, with 30 pairs of 3D chest CT images. To avoid overfitting caused by a small amount of training data, data augmentation is necessary. The 30 pairs of scans were augmented by a 3D TPS transformation method to form the training set, which contained a total of 6060 scans (3030 pairs of scans). As shown in Figure 3, 3D TPS transformation can give the arbitrary nonrigid deformation of the whole space by adjusting the control points of the corresponding positions in the simulated images and the real images. The favorable experimental result on the test set illustrated that the ConvNet trained with augmented data had a strong generalization ability.

The traditional methods generally execute many iterations to realize one registration. Our research is an extension of traditional methods that use deep learning theory. The proposed method can react to traditional image registration as a learning problem. A pair of unseen 3D chest CT images can be aligned in one shot. As shown in Table 2, the execution times of our method were much shorter than those of the traditional methods, which is very important in critical time applications in the real world. These regularizations in the traditional method mean more execution time and more iterations. The regularization terms $R_{\mathrm{jac}}$ and $R_{\mathrm{der}}$ that were used in this study have increased memory consumption, but they had no extra cost to execution time. The qualitative registration performance adopted the absolute different images that are shown in Figure 5. The absolute minimum difference obtained by our method was (*β *=$\;10^{-4}\;$,$10^{-5}$). The quantitative registration results are shown in Figures 4, 6, and Table 1. The proposed method achieved optimal Dice scores. The TRE achieved by our method was close to that achieved by traditional methods. This finding might be a result of the limitations of GPU memory and the suboptimal ConvNet architecture selected. Additionally, ConvNet was sensitive to image contour features and good at image contour registration. Table 2 also provides a comparison between our methods and the traditional methods in the number of folds. The proposed method achieved the minimum number of folds. ConvNet directly penalized the spatial position where the Jacobian deformation was singularity. It is worthwhile to note that our method (*β *=$\;10^{-3}\;$) achieved no folding with promising TRE and Dice scores. For traditional algorithms, achieving strictly positive Jacobian values is a theoretical ideal that is limited by such things as the chosen gradient step and the image resolution, to which all algorithms are susceptible.

The major advantage of the learning‐based method is the ability to master the law of image registration during training. This study provided a comparison between our method and VoxelMorph, both of which are based on unsupervised learning. The experimental results achieved by the proposed method (TRE = 2.09 mm, Dice = 0.987) were superior to VoxelMorph (TRE = 2.35, Dice = 0.980). VoxelMorph used an insufficient technique of smoothing the displacement fields to produce more regions of noninvertibility (folding), which was the reason for the lower TRE. Better invertibility occurs at the expense of registration accuracy. In extensive experiments, a higher Dice score, a lower TRE, and a larger number of folding voxels were obtained when setting a smaller *β* (*β *=$\;10^{-4}\;$,$10^{-5}$). The lower the Dice score was, the higher the TRE, and no folding voxels were achieved when setting a larger *β* (*β *=$\;10^{-2}\;$,$10^{-3}$). This tradeoff was easy to explore by carefully adjusting the size of *β*. Both the proposed method and VoxelMorph used STN to warp the moving images into warped images. This finding indicated that our transform standard was the same as VoxelMorph. VoxelMorph used a simple U‐Net to learn image features. However, many specified multiscale inception models were added in the proposed ConvNet between skip connections to extract features at multiscale levels. The ability of ConvNet to learn highly discriminative features was enhanced by fusing abundant features. Figure 7 provides the representative results. The proposed method achieved a more competitive displacement field (abundant deformation with almost no folding) than VoxelMorph. Figure 4 illustrates that our method could correct large deformations and achieve a small registration error. Additionally, our method had a positive effect on the residual distance in three directions, especially in the Z direction.

This study converted image registration into a learning problem, that is, learning the optimal parameters in the global mapping function. The alignment of the single‐modality image could be driven by minimizing negative NCC, which is based on the intensity image appearance. The first derivative of the displacement and negative Jacobian determinant were used as transformation smoothness constraints. A significant limitation of the proposed method was that the input images must to be resized. The loss of image information could be minimized by resizing the image through trilinear interpolation. Due to the limitation of GPU memory, the size of the image input ConvNet was limited, which might be the main reason for the registration error. In the future, when the memory problem has been addressed, we might use a large ConvNet for full‐size image end‐to‐end training. The proposed method might also support multimodality image registration by replacing NCC with Mutal information.

## 5 CONCLUSIONS

In this study, a ConvNet method based on unsupervised learning for the deformable registration of 3D chest CT images was proposed. Data augmentation based on the 3D TPS transformation method was able to solve the problem of data limitation. The proposed method achieved accurate registration results, with hardly any folding occurring. The proposed method was 2–3 orders of magnitude faster than ANTs (SyN). The method has the potential to be applied in clinical practice.

## FUNDING

This work was supported by the National Natural Science Foundation of China [Grant No. 51775368], National Natural Science Foundation of China [Grant No. 5171101938], and the Technology Planning Project of Guangdong Province, China [Grant No. 2017B020210004].

## CONFLICTS OF INTEREST

The authors declare that they have no conflicts of interest

## ETHICAL APPROVAL

All procedures performed in studies involving human participants were in accordance with the ethical standards of the institutional and/or national research committee and with the 1964 Helsinki declaration and its later amendments or comparable ethical standards.

## INFORMED CONSENT

Informed consent was obtained from all individual participants included in the study.

## AUTHOR CONTRIBUTION

Yongnan Zheng made the conception and design of the study, drafted the article, and revised it critically for important intellectual content. Shan Jiang and Zhiyong Yang made the approval of the version.

## Data Availability

The data that support the findings of this study are openly available in EMPIRE10^24^ at [https://doi.org/10.1109/TMI.2011.2158349], reference number,^24^ DIR‐Lab 4DCT^25^ at [https://doi.org/10.1088/0031‐9155/55/1/018], reference number,^25^ and POPI^26^ datasets at [https://doi.org/10.1118/1.3523619], reference number.^26^
